# Construction of a ceRNA network in glioma and analysis of its clinical significance

**DOI:** 10.1186/s12864-021-08035-w

**Published:** 2021-10-06

**Authors:** Guangdong Liu, Haihong Li, Wenyang Ji, Haidong Gong, Yan Jiang, Guomin Ji, Guangyao Liu

**Affiliations:** 1 Department of Neurosurgery, Hongqi Hospital affiliated to Mudanjiang Medical University, No. 5, Tongxiang Road, Aimin, HeiLongJiang 157000 MuDanJiang, China; 2 Department of Intensive Care Unit, Hongqi Hospital affiliated to Mudanjiang Medical University, MuDanJiang, China

**Keywords:** Glioma, ceRNA, lncRNA, Weighted gene co-expression network analysis, Prognostic model

## Abstract

**Background:**

Glioma is the most common central nervous system tumor with a poor survival rate and prognosis. Previous studies have found that long non-coding RNA (lncRNA) and competitive endogenous RNA (ceRNA) play important roles in regulating various tumor mechanisms. We obtained RNA-Seq data of glioma and normal brain tissue samples from TCGA and GTEx databases and extracted the lncRNA and mRNA expression data. Further, we analyzed these data using weighted gene co-expression network analysis and differential expression analysis, respectively. Differential expression analysis was also carried out on the mRNA data from the GEO database. Further, we predicted the interactions between lncRNA, miRNA, and targeted mRNA. Using the CGGA data to perform univariate and multivariate Cox regression analysis on mRNA.

**Results:**

We constructed a Cox proportional hazard regression model containing four mRNAs and performed immune infiltration analysis. Moreover, we also constructed a ceRNA network including 21 lncRNAs, two miRNAs, and four mRNAs, and identified seven lncRNAs related to survival that have not been previously studied in gliomas. Through the gene set enrichment analysis, we found four lncRNAs that may have a significant role in tumors and should be explored further in the context of gliomas.

**Conclusions:**

In short, we identified four lncRNAs with research value for gliomas, constructed a ceRNA network in gliomas, and developed a prognostic prediction model. Our research enhances our understanding of the molecular mechanisms underlying gliomas, providing new insights for developing targeted therapies and efficiently evaluating the prognosis of gliomas.

**Supplementary Information:**

The online version contains supplementary material available at 10.1186/s12864-021-08035-w.

## Background

Glioma is the most common intracranial primary malignant tumor, accounting for approximately 50% of all brain tumors, with a worldwide incidence rate of 4.67–5.73/100,000 [1, 2]. At present, the treatment of glioma mainly consists of surgical resection combined with postoperative radiotherapy and chemotherapy. However, gliomas are diffusely infiltrating, and because the central nervous system is fragile, various treatments affect its function [3]. Therefore, patients have poor prognosis and quality of life, relapse, and a short survival period. According to the World Health Organization (WHO) Classification of Tumors of the Central Nervous System, grade II and III gliomas are mainly composed of low-grade gliomas (LGG), while grade IV of glioblastoma (GBM). In some patients with LGG, the disease progresses slowly, while in others, it may transform into GBM. Despite multimodal treatment strategies, including surgery, chemotherapy, and radiotherapy, prognosis of GBM remains poor with a median survival time of 12–14 months and 5-year survival rate of approximately 5% [4, 5]. Therefore, in-depth studies on the pathogenesis of glioma to explore potential molecular markers and therapeutic targets to improve diagnosis and treatment are crucial.

In the human genome, about 90% of the genes get transcribed, about 2% encode proteins, and the remaining are transcribed into non-coding RNA (ncRNA) [6]. Long non-coding RNA (lncRNA) is an ncRNA with a length of more than 200 nucleotides, which exists mainly in the nucleus, has certain tissue specificity, and poor interspecies sequence conservation [7]. LncRNA can participate in epigenetic regulation, chromatin modification, transcription interference, and other biological processes, and can thus regulate processes such as the cell cycle, cell differentiation, proliferation, apoptosis, and invasion [8–10]. It is evident that some lncRNAs having carcinogenic or tumor suppression effects and participate in a variety of tumor molecular mechanisms, which can be used as molecular biomarkers of cancer [11]. H19 is an lncRNA whose corresponding gene is located on chromosome 11. In glioma, when the expression of H19 increases, the expression of miR-152 decreases; consequently, the downregulation of H19 can negatively regulate the expression of miR-152 to inhibit the proliferation and invasion of glioma cells [12]. Another lncRNA, NEAT1, promotes the occurrence and development of gliomas by regulating the miR-185-5p/DNMT1/mTOR signaling pathway [13]. Previous studies have found that the upregulation of lncRNA SNHG16 expression is related to the decreased survival rate of some tumors, such as glioma and digestive and urinary system tumors [14]. LncRNA CASC2 can inhibit tumor cell proliferation and induce apoptosis in some tumors, including glioma, breast cancer, and colorectal cancer [15]. Furthermore, in glioma, lncRNA SLC26A4-AS1 is underexpressed and promotes the transcriptional activity of NPTX1 by recruiting NFĸB1, thereby exerting an anti-angiogenic effect [16]. Thus, lncRNAs have a variety of roles in different tumors, with increasing research on their role in gliomas.

Competitive endogenous RNAs (ceRNAs) play an important role in the molecular regulation of tumors by modulating mRNA expression via the development of a new type of regulatory relationship between lncRNA and miRNA [17]. The lncRNA NCK1-AS1 upregulated in glioma can act as a ceRNA on miR-138-2-3p to upregulate the expression of TRIM24, thereby activating the Wnt/β-catenin pathway [18]. Moreover, LINC00473 can act as a ceRNA on miR-637 to regulate CDK6 expression and have cancer-promoting effects on glioma [19], whereas lncRNA MATN1-AS1 can have ceRNA-like effects on miR-200b/c/429 to increase CHD1 expression, thereby promoting the progression of glioma [20]. Zhu et al. constructed a specific ceRNA network composed of lncRNAs, miRNAs, and mRNAs in gliomas and analyzed the survival of lncRNAs, showing that lncRNAs can be used as potential biomarkers for predicting the prognosis of patients with glioma; however, there were only five normal samples used in their study [21]. Our study included 1146 normal brain tissue samples and 5 adjacent tissue samples, which were combined with weighted gene co-expression network analysis (WGCNA) for comprehensive analysis.

**Fig. 1:**
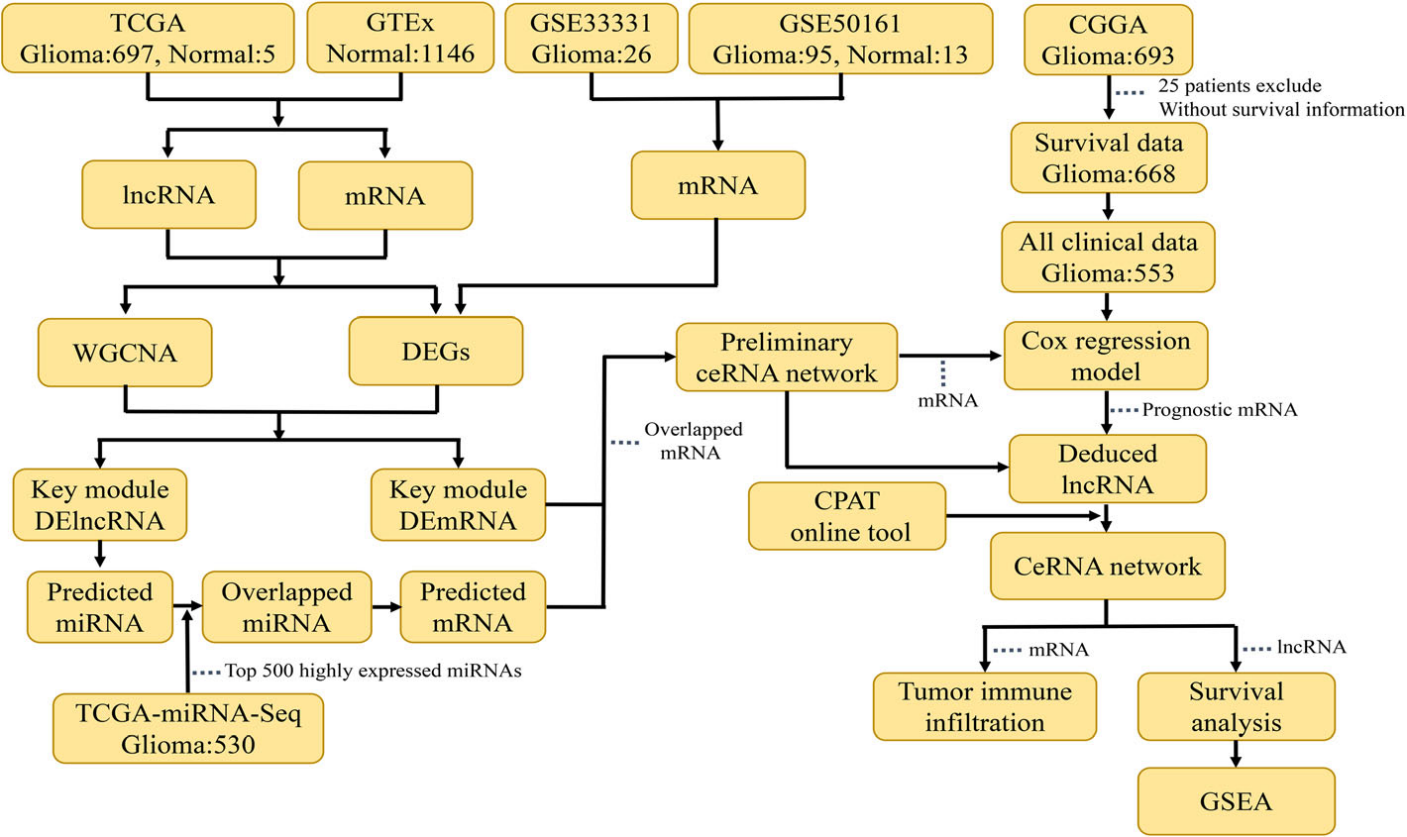
Study workflow. TCGA, The Cancer Genome Atlas; GTEx, Genotype Tissue Expression; GEO, Gene Expression Omnibus; CGGA, Chinese Glioma Genome Atlas; WGCNA, Weighted Gene Co-expression Network Analysis; DEG, differentially expressed genes; GSEA, Gene Set Enrichment Analysis

In this study, we carried out WGCNA and differential expression analysis based on The Cancer Genome Atlas (TCGA) and Genotype Tissue Expression (GTEx) databases to identify the differential lncRNAs and mRNAs that are highly correlated to glioma. The differential analysis was carried out using mRNA data from the Gene Expression Omnibus (GEO) database and the differential mRNAs obtained from the combined data of TCGA and GTEx were further screened. These mRNAs were used to perform a Cox regression analysis based on the Chinese Glioma Genome Atlas (CGGA) database and develop a prognostic prediction model for glioma patients. Finally, we constructed a ceRNA network and performed survival and functional analyses on the newly discovered lncRNA in relation to glioma using the network. Figure 1 shows the flow diagram of our study. Our research paves the way for further studies by shedding light on the novel mechanisms of glioma, as well as the potential molecular markers and therapeutic targets for glioma treatment.

## Results

### DElncRNAs and DEmRNAs

From the TCGA and GTEx combined dataset, we identified differentially expressed (DE) lncRNAs and mRNAs, including 194 upregulated and 280 downregulated lncRNAs, and 2223 upregulated and 2334 downregulated mRNAs. From the GEO combination dataset, we identified 2479 upregulated and 2056 downregulated mRNAs. We integrated DEmRNAs that overlapped between the aforementioned datasets and identified 828 upregulated and 716 downregulated mRNAs (*p* < 0.05, |log2 fold change (FC)| ≥ 1).

### WGCNA reveals the key module of glioma

**Fig. 2:**
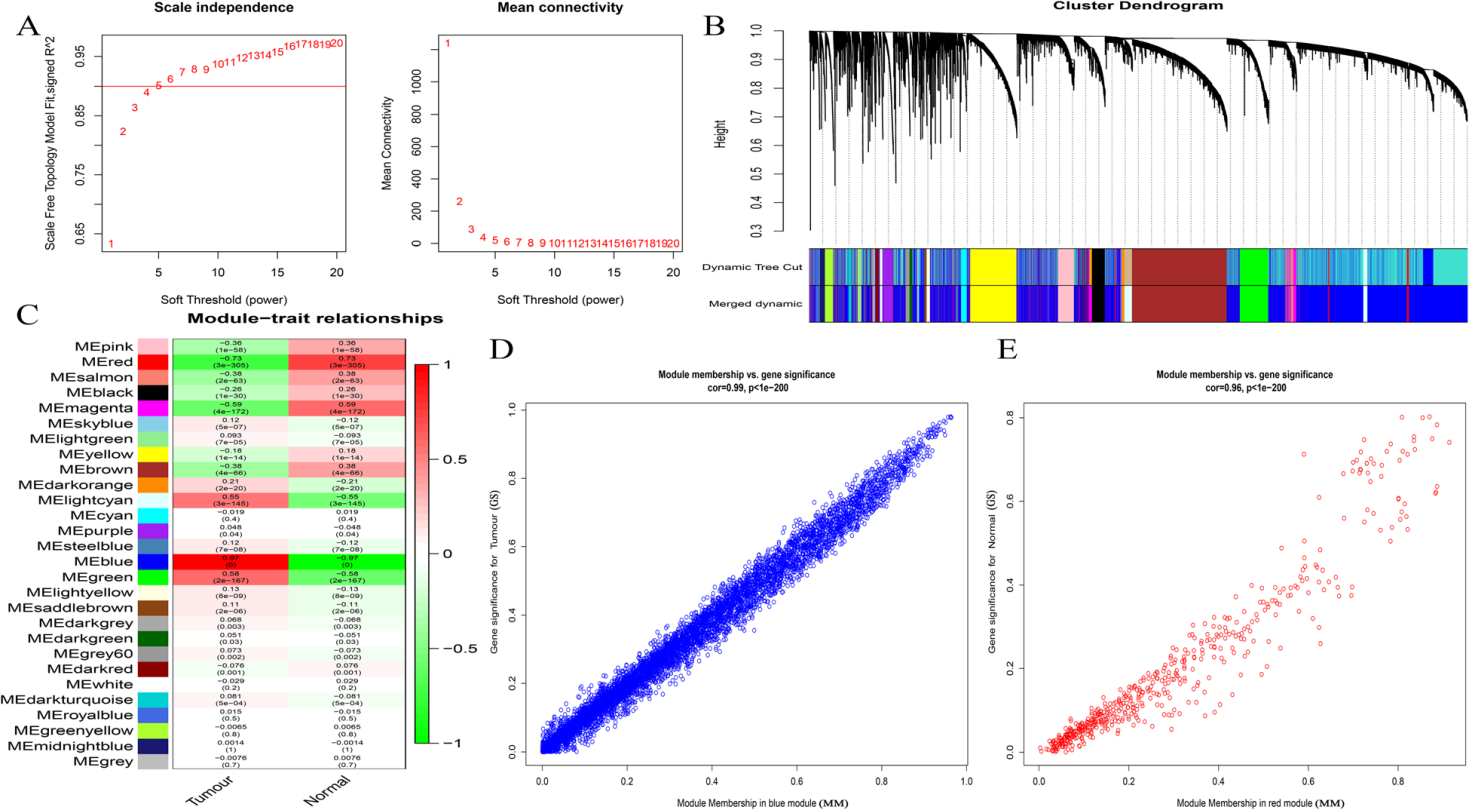
Identification of lncRNAs modules highly related to traits. **A** Determination of soft-thresholding power in the lncRNAs WGCNA. Left: Analysis of the scale-free fit index for various soft thresholding powers (β). Right: Analysis of the mean connectivity for various soft-thresholding powers. **B** Cluster dendrogram of all lncRNA in the co-expression network. **C** Module-trait associations of lncRNAs were evaluated by correlations between MEs and clinical traits. **D**-**E** The correlation between gene significance (GS) and module membership (MM) in the blue and red modules

We performed WGCNA of the combined TCGA and GTEx datasets, constructed a co-expression network for all the lncRNAs (including 15,239 lncRNAs), and determined that the β value in the network was 3 (Fig. 2a). In addition, a dynamic tree cutting method was used to generate co-expression modules, and the closely related modules were merged into larger modules. Finally, 28 modules were generated in the lncRNA co-expression network (Fig. 2b). The module eigengenes (ME) of the blue and red modules had the strongest correlations with the tumor and normal traits, respectively (Fig. 2c). Figures 2d-e show the correlation between gene significance and module membership. The blue and red modules are the key modules, containing 7976 lncRNAs.

**Fig. 3:**
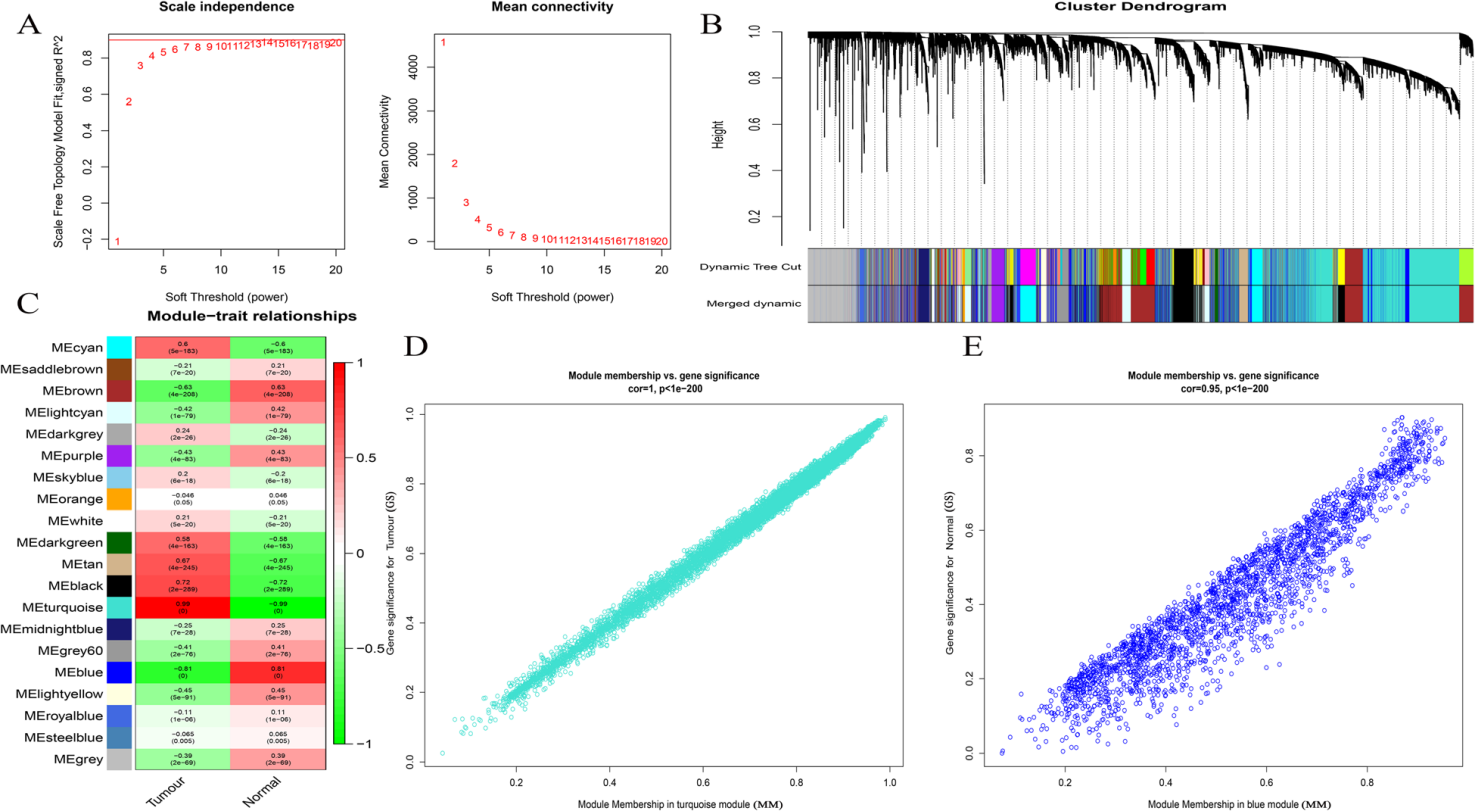
Identification of mRNAs modules highly related to traits in the combined data of TCGA and GTEx. **A** Determination of soft-thresholding power. **B** Cluster dendrogram of all mRNA in the co-expression network. **C** The correlation between modules and traits were displayed. **D**-**E** The correlation between gene significance (GS) and module membership (MM) in the turquoise and blue modules

Further, we constructed a co-expression network of all the mRNAs based on the combined data from TCGA and GTEx, including 20,234 mRNAs. The β value in the co-expression network was 7 (Fig. 3a), and a total of 20 modules were generated (Fig. 3b). The ME of the turquoise and blue modules had the strongest correlations with the tumor and normal traits, respectively (Fig. 3c). Figures 3d–e show the correlation between gene significance and module membership. The turquoise and blue modules are considered key modules and contain 8169 mRNAs.

### Preliminary construction of ceRNA network

Through WGCNA of the lncRNAs, we obtained 7976 lncRNAs in the blue and red modules and integrated this with DElncRNAs to obtain 395 overlapping lncRNAs. The overlapping lncRNAs were then used to predict the miRNAs. In addition, combining the predicted miRNAs with the first 500 highly expressed miRNAs downloaded from TCGA database, 59 miRNAs were obtained. Next, 151 target mRNAs were predicted by 59 miRNAs. The predicted mRNAs, key modules of WGCNA, and DEmRNAs were then analyzed, and we obtained eight mRNAs (one downregulated and seven upregulated mRNAs). Finally, these eight mRNAs were used to analyze and screen the above information, and 25 lncRNAs and two miRNAs were obtained (Fig. 8a).

### Cox proportional hazards regression model

**Fig. 4:**
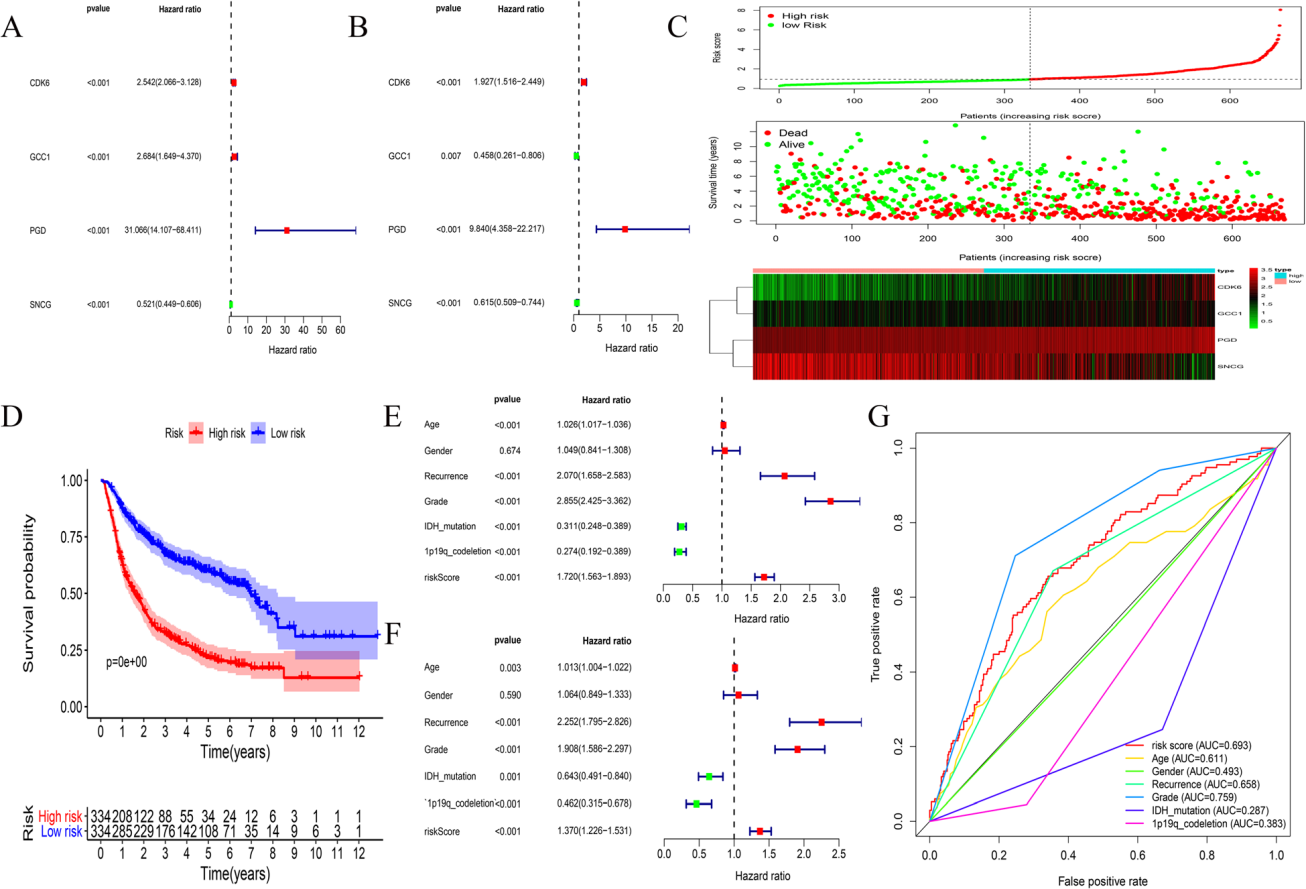
4-mRNA prognosis model establishment and verification. **A**-**B** Univariate Cox regression analysis and multivariate Cox regression analysis were used to construct prognostic models. **C** Correlation between the prognostic signature and the overall survival of patients. The distribution of risk scores (upper), survival time (middle) and lncRNA expression levels (below). The red dots and lines represent the patients in high-risk groups. The green dots and lines represent the patients in low-risk groups. **D** Kaplan-Meier survival curves of overall survival among risk score groups. **E** Univariate Cox regression analyses of clinical factors associated with overall survival. **F** Multivariate Cox regression analyses of clinical factors associated with overall survival. **G** Receiver operating characteristic (ROC) curve related to clinical factors

The CGGA dataset (*n* = 668) was used to construct a regression model of glioma patients. Univariate Cox regression analysis was performed on the eight previously obtained mRNAs, and four mRNAs related to prognosis were identified (*p* < 0.001) (Fig. 4a). Next, a multivariate Cox analysis was used to construct a Cox proportional hazards regression model for glioma patients (Fig. 4b). According to the median risk score, all the patients were divided into two groups, namely, the high-risk and low-risk groups. Figure 4c shows the survival status, survival time, and mRNA expression levels of the patients. Survival analysis showed that the patients in the low-risk group had a longer survival time (*p* < 0.001) compared to those in the high-risk group (Fig. 4d). Univariate Cox regression analysis showed that age, recurrence, glioma grade, isocitrate dehydrogenase (IDH) mutation, 1p19q co-deletion, and risk score were survival predictors in the CGGA dataset (*n* = 553) (*p* < 0.001) (Fig. 4e). Multivariate Cox regression analysis showed that risk score, age, recurrence, glioma grade, IDH mutation, and 1p19q co-deletion were independent risk factors (Fig. 4f) (*p* < 0.005). The AUC values of the ROC curve for risk score, recurrence, and grade were 0.693, 0.658, and 0.759, respectively (Fig. 4g). Thus, we developed a prognostic prediction model for glioma patients using four mRNAs, namely, SNCG, PGD, CDK6, and GCC1.

### Relationship between key mRNAs and tumor immune infiltration

**Fig. 5:**
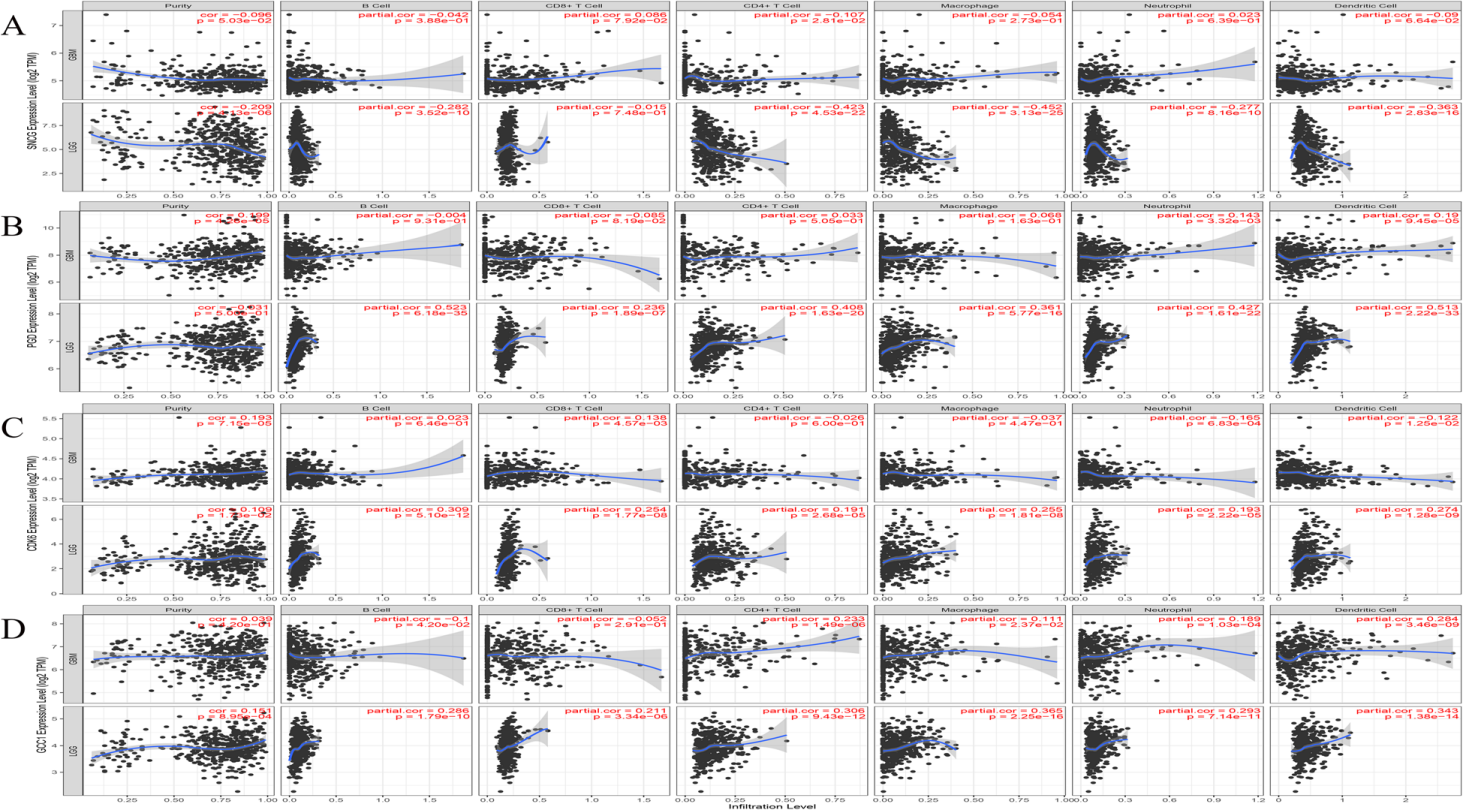
The relationship between mRNA expression and immune infiltration in glioma. **A** SNCG; **B** PGD; **C** CDK6; **D** GCC1. *P* < 0.05 denotes significance

The tumor microenvironment is mainly composed of the tumor, stromal, and immune cells. We analyzed and visualized the relationship between four kinds of mRNAs (SNCG, PGD, CDK6, and GCC1) and tumor purity and immune cell infiltration using the TIMER tool (Fig. 5). In LGG, SNCG is significantly related to CD4^+^ T cells (cor = − 0.423), macrophages (cor = − 0.452), dendritic cells (cor = 0.363); PGD is significantly related to B cells (cor = 0.523), dendritic cells (cor = 0.513), and neutrophils (cor = 0.427); CDK6 is related to B cells (cor = 0.309), CD8^+^ T cells (cor = 0.254) and dendritic cells (cor = 0.274); and GCC1 is related to CD4^+^ T cells (cor = 0.306), dendritic cells (cor = 0.343), and macrophages (cor = 0.365) (all data *p* < 0.0001). We found that they are associated with immune infiltration in GBM and LGG, and that the correlation is especially strong in LGG.

### Screening the ceRNA network

We used four mRNAs obtained from multiple Cox analysis to screen the ceRNA network and obtained two miRNAs and 25 lncRNAs. The CPAT online tool was used to verify lncRNA, 21 of which had no coding ability. Cytoscape was used to visualize the ceRNA network of 21 lncRNAs, two miRNAs, and four mRNAs (Fig. 8b).

### LncRNA related to patient survival

**Fig. 6:**
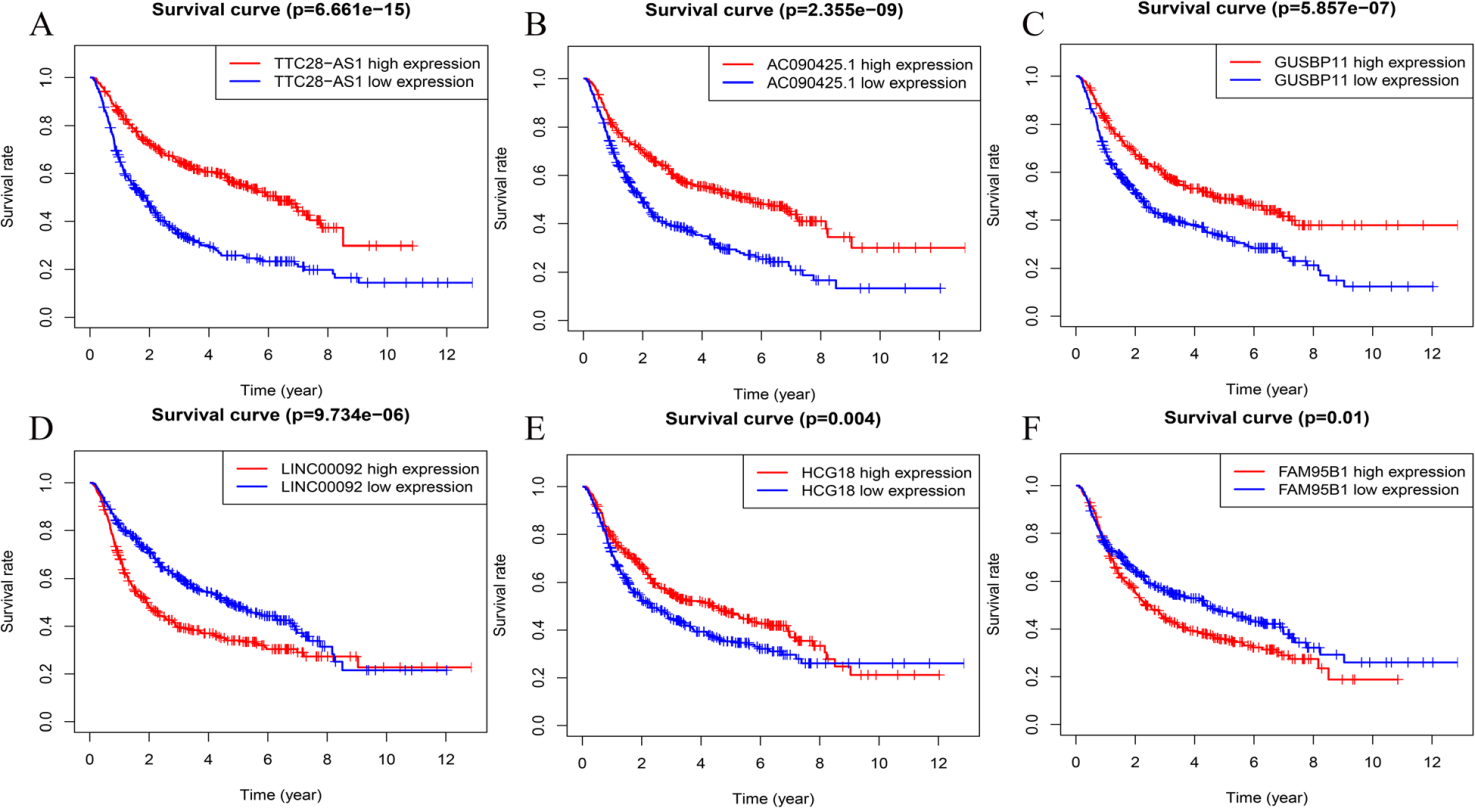
Kaplan–Meier survival curves of six lncRNAs in the ceRNA network. **A** TTC28-AS1; **B** AC090425.1; **C** GUSBP11; **D** LINC00092; **E** HCG18; **F** FAM95B1. *P* < 0.05

In the CGGA database (*n* = 668), Kaplan–Meier analysis was used to verify the correlation between lncRNAs and the overall survival (OS) of glioma patients. In the Kaplan–Meier analysis of 21 lncRNAs, 12 lncRNAs were found to be closely related to the OS of glioma patients. Among these, there were six lncRNAs (TTC28-AS1, AC090425.1, GUSBP11, LINC00092, HCG18, and FAM95B1) that have not been studied in gliomas, and we visualize the Kaplan–Meier analysis results (*p* < 0.05) (Fig. 6).

### GSEA

**Fig. 7:**
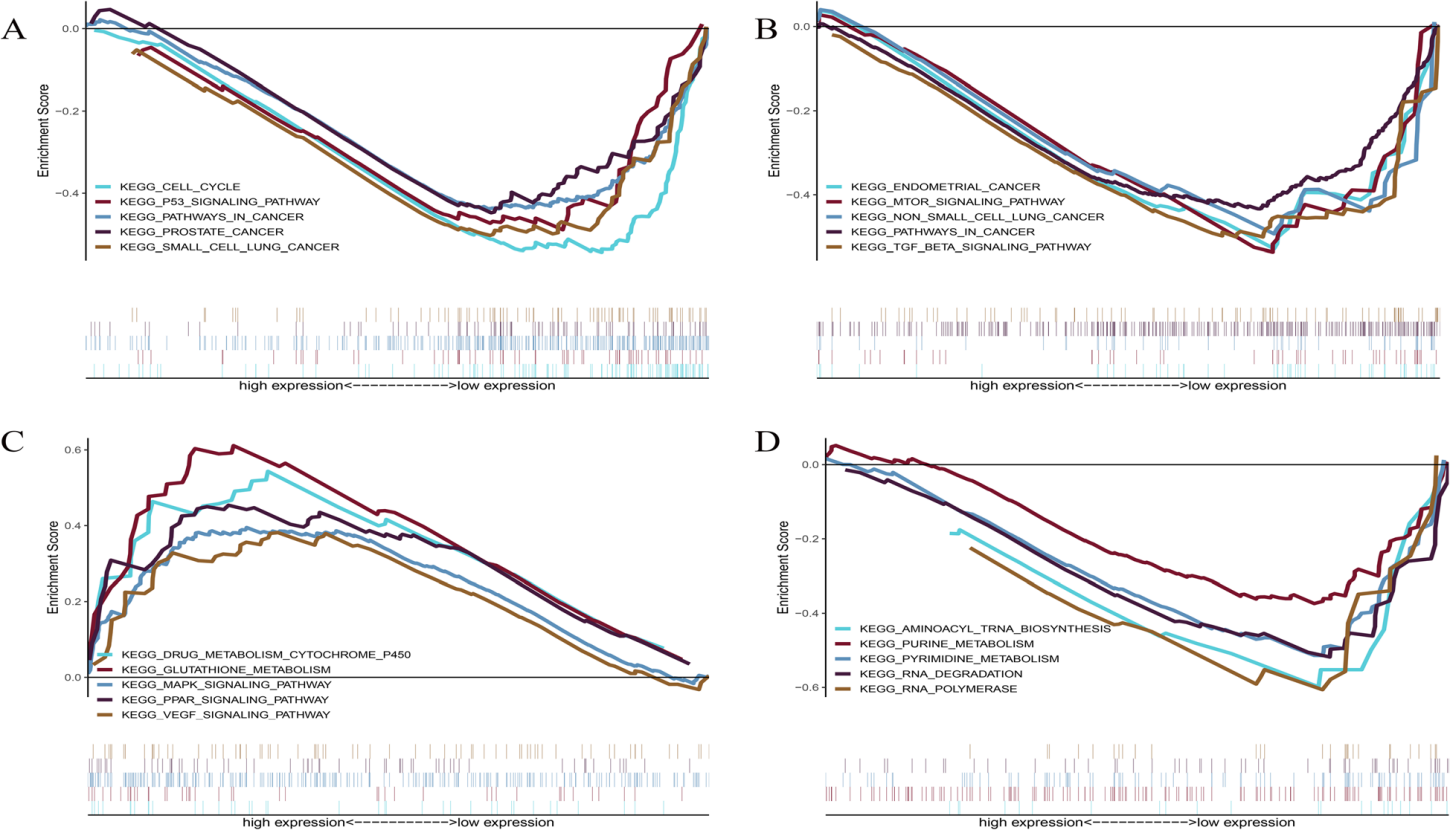
GSEA is used for signal pathway analysis based on CGGA database. A total of 693 samples were divided into two groups based on the median expression level. **A** AC090425.1; **B** HCG18; **C** LINC00092; **D** TTC28-AS1. *P* < 0.05

Using the CGGA database (*n* = 693), we performed GSEA analysis on the six lncRNAs identified previously and showed that four were closely related to cancer. AC090425.1 shows potential effects on the p53 signaling pathway, small cell lung cancer, cell cycle, and other pathways involved in cancer (Fig. 7a). HCG18 showed potential effects on pathways in cancer, non-small cell lung cancer, endometrial cancer, and mTOR signaling pathway (Fig. 7b). LINC00092 was found to be closely related to the drug metabolism cytochrome p450, glutathione metabolism, and the VEGF signaling pathway (Fig. 7c). Lastly, TTC28-AS1 was found to be associated with RNA polymerase, RNA degradation, and aminoacyl tRNA biosynthesis (Fig. 7d).

### CeRNA network in glioma

**Fig. 8:**
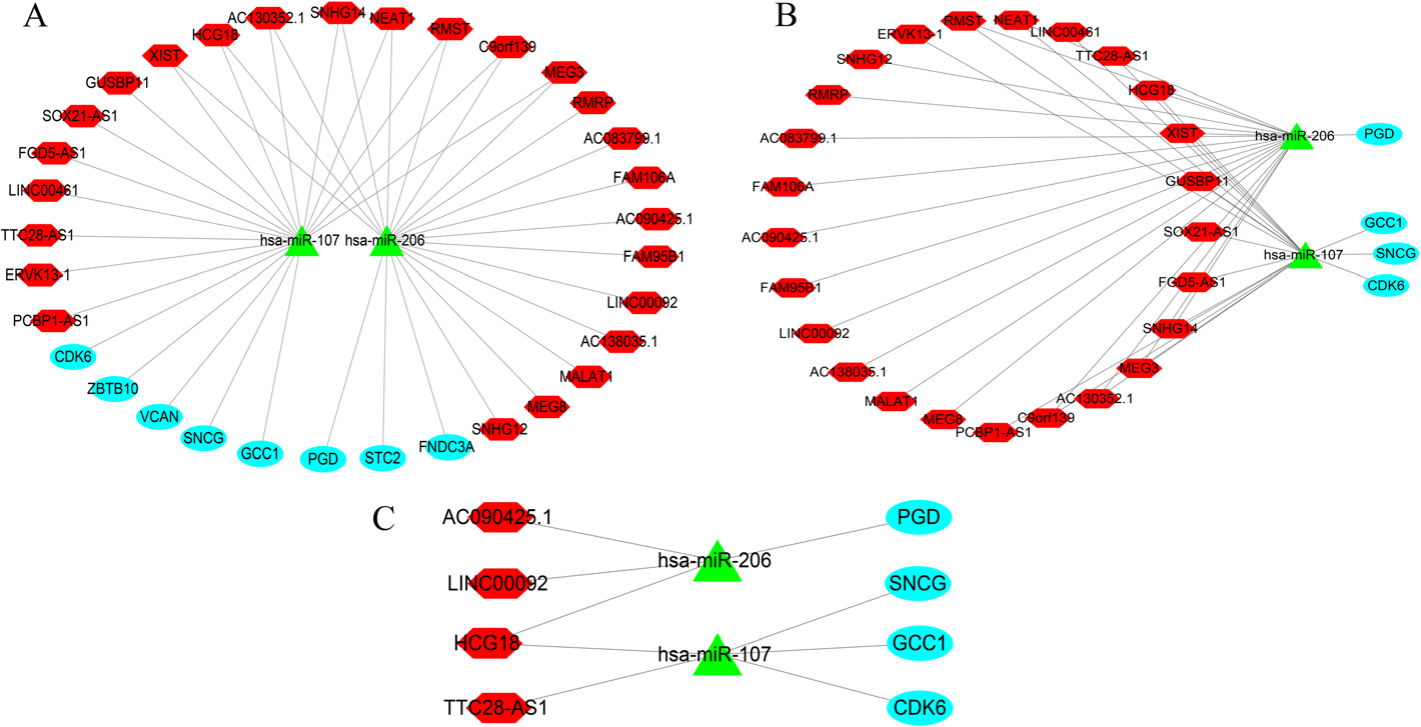
Visualize ceRNA network relationships. Notes: Red hexagon denotes lncRNA, green triangle represents miRNA, and cyan ellipse represents mRNA

After a series of analyses, we finally obtained a ceRNA network composed of four lncRNAs, two miRNAs and four mRNAs (Fig. 8c).

## Discussion

Gliomas are the most aggressive primary tumors in the central nervous system accounting for approximately 50 to 60% of primary intracranial tumors [22]. In recent years, their incidence has increased gradually [23]. Gliomas often spread and invade the surrounding normal brain tissue and are insensitive to radiotherapy and chemotherapy; further, the prognosis of patients is poor. Understanding the molecular mechanisms of tumor activity is important for tumor diagnosis, treatment, and prognosis evaluation. LncRNAs may act as oncogenes or tumor suppressor genes in cancer and regulate tumors through various mechanisms. The ceRNA regulatory network between lncRNAs, miRNAs, and mRNAs is a newly discovered regulatory relationship. Moreover, the ceRNA network plays an important role in modulating the biological characteristics of cancer.

In this study, based on WGCNA and differential expression analysis, we constructed a ceRNA network containing 21 lncRNAs, two miRNAs, and four mRNAs. Using univariate and multivariate Cox regression analysis, a Cox proportional hazard regression model with four key mRNAs, SNCG, PGD, CDK6, and GCC1, was constructed. We calculated the patients’ risk score and verified that the prognostic model has good performance status. We found the risk score, age, recurrence, glioma grade, IDH mutation, and 1p19q co-deletion were independent risk factors in glioma. Moreover, these four mRNAs were found to be closely related to the immune infiltration of LGG. In the ceRNA network, there were 12 lncRNAs related to survival. Based on a thorough literature review, out of these 12 lncRNAs, we selected six that have not been previously studied in gliomas for GSEA analysis. We found four lncRNAs that were directly or indirectly related to a variety of cancers, namely, TTC28-AS1, AC090425.1, LINC00092, and HCG18. We found that AC090425.1 and HCG18 were related to a variety of cancer-related pathways, while TTC28-AS1 and LINC00092 were related to diverse cancer functions. Briefly, AC090425.1 may be important in the p53 signaling pathway and cell cycle, while HCG18 in the regulation of the mTOR signaling pathway. After a series of screening, we finally obtained a ceRNA network composed of four lncRNAs, two miRNAs and four mRNAs.

High expression of SNCG is related to poor prognosis of ovarian, breast, and endometrial cancers, and to the aggressiveness of ovarian and endometrial cancers [24–26]. In breast cancer, SNCG enhances the immunosuppressive effect by inhibiting differentiation and activation of dendritic cells [27]. Although PGD can effectively block the proliferation of hereditary leiomyomatosis and renal cell carcinoma cells in vitro and in vivo, it can promote metastasis of pancreatic cancer [28, 29]. CDK6 is a key regulatory factor in the cell cycle and a cancer-promoting factor in gliomas. It negatively correlates with patient survival and low expression of CDK6 can significantly enhance the sensitivity of gliomas to chemotherapy [30, 31]. CDK4/6 inhibitors can significantly enhance T cell activation and inhibit the proliferation of regulatory T cells [32, 33]. Unfortunately, there are no tumor-related reports on GCC1. Conclusively, the aforementioned findings are similar to that of the present study. In addition, we also found that SNCG and CDK6 are related to prognosis of glioma; SNCG to dendritic cells; and CDK6 to immunity.

The microRNAs in the ceRNA network we obtained have been studied a lot, and they have a clear effect on gliomas. Reportedly, miR-206, which is underexpressed in gliomas, has a tumor suppressor effect, is related to the survival time of patients, and is a potential biomarker [34, 35]. Further, miR-107, which is also underexpressed in gliomas, supposedly regulates glioma development by serving as ceRNA of LINC00662 and LINC00152 [36, 37]. In conclusion, miR-206 and miR-107 play important roles in glioma.

HCG18 is overexpressed in gastric cancer and indirectly regulates Hippo signal transduction activity by inhibiting the expression of miR-141-3p [38]. It is also overexpressed in colorectal cancer tissues and cell lines and can enhance MTDH/Wnt/β-catenin signaling through the sponging of miR-1271, thereby exerting potential carcinogenic effects [39]. It can be used as a ceRNA of miR-140 to increase the expression of CCND1, thus playing a role in the Wnt/β-catenin signaling pathway to promote the development of nasopharyngeal cancer [40].

Some studies have shown that the low expression of TTC28-AS1 may be related to the occurrence of type 2 diabetes; however, its function in tumors has not yet been studied [41]. LINC00092 is overexpressed in metastatic ovarian cancer and is related to the invasion and migration of ovarian cancer cells and poor prognosis; in addition, it can also be used as a diagnostic molecular biomarker for colon adenocarcinoma [42, 43]. Lastly, there is hardly any relevant research on AC090425.1 conducted so far.

## Conclusion

We constructed a glioma lncRNA–miRNA–mRNA ceRNA network, in which four lncRNAs that have not been previously studied in gliomas can be used as new potential therapeutic targets. In addition, these four mRNAs in the ceRNA network constitute a reliable prognostic prediction model, and they are related to the immune infiltration of LGG. Our study combined several glioma and normal brain tissue samples from multiple databases for a comprehensive analysis. However, the lack of cell or animal models for the verification of our data is a limitation of our study. Ultimately, our research enhances our understanding of the molecular mechanisms underlying glioma, and thus provide new insights for developing targeted therapies and efficiently evaluating the prognosis of glioma.

## Materials and methods

### Data resource

In this study, we comprehensively analyzed the data from four different databases. First, we used the UCSC Xena Browser (https://xena.ucsc.edu/) to access the RNA-Seq data from the GTEx and TCGA (LGG + GBM) databases. The former contains 702 samples, comprising 697 glioma and five adjacent tissue samples, and the latter contains 1146 normal brain tissue samples. We downloaded miRNA-Seq data of 530 glioma samples from TCGA databases. Next, we obtained two datasets, GSE33331 (comprising 26 glioma samples; platform: Affymetrix-GPL570) and GSE50161 (comprising 13 normal and 117 brain tumor samples; platform: Affymetrix-GPL570) from the GEO database (https://www.ncbi.nlm.nih.gov/geo/). The GSE50161 dataset was screened for glioma samples, and 95 glioma and 13 normal brain tissue samples were obtained. Finally, we downloaded the RNA-Seq and clinical data of 693 glioma samples from the CGGA database (http://cgga.org.cn/), of which 668 patients had survival information and 553 patients had complete clinical information.

### Data preprocessing

The RNA-Seq data extracted from TCGA and GTEx databases were converted into gene symbols according to GENCODE (https://www.gencodegenes.org/human/). The R package oligo [44] was used for format conversion, missing data filling, background correction, and data normalization. RNA expression level of all the samples was presented as log2 (fpkm + 1), and genes expressed as 0 were removed. Furthermore, the expression matrices of mRNA and lncRNA were extracted from TCGA and GTEx combined data. We also annotated the GEO data through Affymetrix-GPL570 platform annotation files, which were combined and normalized to obtain an mRNA expression matrix. Lastly, we calibrated and normalized the CGGA data to perform log2 transformation on all the gene expression data.

### Screening of DEGs

For the combined data of TCGA and GTEx, the Limma R package [45] was used to screen the differentially expressed genes (DEGs), particularly the differentially expressed lncRNAs (DElncRNAs). We also performed differential expression analysis on the mRNA data from the combined GTEx and TCGA datasets and the combined GEO dataset, and further screened the differential genes shared by the two datasets to obtain differentially expressed mRNAs (DEmRNAs). Values of *p* < 0.05 and |log2 FC| ≥ 1 were considered to have significant differences and were selected.

### WGCNA

Based on the combined data from TCGA and GTEx, we performed WGCNA on lncRNA and mRNA data, respectively. WGCNA R package [46] was used to construct a co-expression network and find modules related to the clinical features of glioma. First, hierarchical cluster analysis was performed to check the heterogeneity of the sample and the best soft threshold power (β) was selected according to the scale-free topology standard. Next, a weighted adjacency matrix was constructed and transformed into a topological overlap matrix. Finally, a weighted gene co-expression network was constructed, and a dynamic tree cutting algorithm was used to determine the different gene modules. To assess the correlation between the module and glioma, the relationship between module membership (MM) and gene significance (GS) was calculated and scatter plots were used to show the correlation between glioma and key gene modules.

### Prediction of the lncRNA–miRNA–mRNA relationship

The lncRNA key module and DElncRNA comprehensive analysis were used to screen for overlapping lncRNAs. The miRcode database (http://www.mircode.org/) was used to predict the interactions between lncRNA and miRNA. We analyzed the predicted miRNA and the top 500 highly expressed miRNA data obtained from TCGA database to obtain the overlapping miRNAs. The miRTarBase (http://mirtarbase.mbc.nctu.edu.tw/), miRDB (http://www.mirdb.org/), and TargetScan (http://www.targetscan.org/) were used to predict the target mRNA of miRNA and were predicted by the three databases simultaneously. We analyzed the key mRNA modules obtained using WGCNA and predicted mRNA and DEmRNA to obtain overlapping mRNA. Finally, the prediction results were evaluated to obtain the ceRNA relationship of the lncRNA–miRNA–mRNA.

### Construction of the cox regression model

We initially obtained the ceRNA network relationship and screened the network using the Survival R package to perform univariate Cox regression analysis on the mRNAs obtained from the CGGA data. Next, a multivariate Cox regression analysis was used to design a prognostic prediction model and determine the corresponding prognostic characteristic coefficient of glioma. In addition, we calculated risk scores to predict survival time, using the median value of the risk scores to divide the samples into high-risk and low-risk groups. The “predict ()” function was used to calculate the risk score as follows: risk score = h0(t) × exp.(β1x1 + β2x2 + … + βnxn). The clinical factors were then analyzed through univariate and multivariate Cox analyses, and the correlation between the prognostic characteristics and overall survival rate of the patient was calculated. Finally, we used the R package SurvivalROC to draw the ROC curve and calculated the area under the curve (AUC) to assess the performance of the prognostic prediction model.

### Tumor-infiltrating immune cell analysis

We used the online tool TIMER (https://cistrome.shinyapps.io/timer/) [47] to study the correlation between key mRNAs and tumor immune cells, including B cells, CD8^+^ T cells, CD4^+^ T cells, macrophages, neutrophils, and dendritic cells.

### Construction of ceRNA network

Based on the mRNAs obtained from the multivariate Cox regression analysis and the predicted lncRNA–miRNA–mRNA relationship, the corresponding miRNAs and lncRNAs were screened, and the CPAT online tool (http://lilab.research.bcm.edu/cpat/index.php) was used to verify the coding ability of lncRNA, thereby constructing a ceRNA network. Cytoscape 3.8.0 software (https://cytoscape.org/) [48] was used to draw the obtained ceRNA network.

### Survival analysis

Kaplan–Meier survival analysis of lncRNAs was performed based on the survival information of 668 patients obtained from the CGGA database. The Survival R package was used to draw the survival curve of the lncRNAs in the ceRNA network and perform a log-rank test (*p* < 0.05).

### Gene set enrichment analysis (GSEA)

The 693 glioma samples, whose data were obtained from the CGGA database, were divided into high and low expression groups based on the median expression levels of the target lncRNA. The lncRNAs related to patient survival in the ceRNA network were analyzed using the GSEA software (http://software.broadinstitute.org/gsea/index.jsp) [49] (*p* < 0.05). The reference gene set “c2.cp.kegg.v7.2.symbol.gmt” was downloaded from MSigDB (https://www.gsea-msigdb.org/gsea/msigdb).

## Supplementary Information

**Additional file 1: Table S1.** In the context of WHO classification, Kaplan-Meier analysis data of 12 lncRNAs.

**Additional file 2: Table S2.** DEmRNA related data.

**Additional file 3: Table S3.** CPAT verifies the coding ability of lncRNA.

**Additional file 4: Table S4.** Kaplan-Meier analysis data of 21 lncRNAs.

**Additional file 5: Table S5.** GSEA analysis results.

**Additional file 6: Table S6.** COX model related data.

**Additional file 7: Table S7.** Figure 4 Related analysis data.

## Data Availability

The data comes from TCGA, GTEx, GEO, CGGA databases, which are all public open platforms. UCSC Xena Browser (https://xena.ucsc.edu/); TCGA (https://portal.gdc.cancer.gov/); GTEx (https://gtexportal.org/home/); GEO (https://www.ncbi.nlm.nih.gov/geo/); CGGA (http://www.cgga.org.cn/); miRcode (http://www.mircode.org/); miRTarBase (http://mirtarbase.mbc.nctu.edu.tw/); miRDB (http://www.mirdb.org/); TargetScan (http://www.targetscan.org/); TIMER (https://cistrome.shinyapps.io/timer/); CPAT (http://lilab.research.bcm.edu/cpat/index.php).

## Abbreviations

LncRNA: Long non-coding RNA
ceRNA: competitive endogenous RNA
TCGA: The Cancer Genome Atlas
NCBI: National Center for Biotechnology Information
GEO: Gene Expression Omnibus
CGGA: Chinese Glioma Genome Atlas
GTEx: Genotype Tissue Expression
WGCNA: Weighted gene co-expression network analysis
GSEA: Gene Set Enrichment Analysis
